# Ethanol inhibits LPS-induced signaling and modulates cytokine production in peritoneal macrophages in vivo in a model for binge drinking

**DOI:** 10.1186/1471-2172-10-49

**Published:** 2009-09-18

**Authors:** Stephen B Pruett, Ruping Fan

**Affiliations:** 1 Department of Cellular Biology and Anatomy, Louisiana State University Health Sciences Center, Shreveport, LA 71130, USA; 2 Department of Basic Sciences, College of Veterinary Medicine, Mississippi State University, Mississippi State, MS 39762, USA

## Abstract

**Background:**

Previous reports indicate that ethanol, in a binge drinking model in mice, inhibits the production of pro-inflammatory cytokines in vivo. However, the inhibition of signaling through TLR4 has not been investigated in this experimental model in vivo. Considering evidence that signaling can be very different in vitro and in vivo, the present study was conducted to determine if effects of ethanol on TLR4 signaling reported for cells in culture or cells removed from ethanol treated mice and stimulated in culture also occur when ethanol treatment and TLR4 activation occur in vivo.

**Results:**

Phosphorylated p38, ERK, and c-Jun (nuclear) were quantified with kits or by western blot using samples taken 15, 30, and 60 min after stimulation of peritoneal macrophages with lipopolysaccharide in vivo. Effects of ethanol were assessed by administering ethanol by gavage at 6 g/kg 30 min before administration of lipopolysaccharide (LPS). Cytokine concentrations in the samples of peritoneal lavage fluid and in serum were determined at 1, 2, and 6 hr after lipopolysaccharide administration. All of these data were used to measure the area under the concentration vs time curve, which provided an indication of the overall effects of ethanol in this system. Ethanol suppressed production of most pro-inflammatory cytokines to a similar degree as it inhibited key TLR4 signaling events. However, NF-κB (p65) translocation to the nucleus was not inhibited by ethanol. To determine if NF-κB composed of other subunits was inhibited, transgenic mice with a luciferase reporter were used. This revealed a reproducible inhibition of NF-κB activity, which is consistent with the observed inhibition of cytokines whose expression is known to be NF-κB dependent.

**Conclusion:**

Overall, the effects of ethanol on signalling in vivo were similar to those reported for in vitro exposure to ethanol and/or lipopolysaccharide. However, inhibition of the activation of NF-κB was not detected as translocation of p65 to the nucleus but was detected using transgenic reporter mice. The observation that ethanol given 24 hr before dosing with LPS modulated production of some cytokines indicates a persistent effect which does not require continued presence of ethanol.

## Background

Acute ethanol exposure can suppress signaling through toll-like receptors (TLR). This signaling normally leads to the production of pro-inflammatory cytokines [1-3]. However, studies of this matter have been performed using macrophage-like cell lines or human monocytes following in vitro exposure to ethanol and an inflammatory stimulus, or using macrophages from ethanol-treated mice stimulated ex vivo. A number of studies in the past several years demonstrate that signaling can be remarkably different in vitro (or ex vivo) and in vivo, even when using the same stimulus [4-6]. In addition, we found that the pattern of IL-10 and IL-12 production was very different in a macrophage-like cell line in vitro and in the peritoneal cavity or serum in vivo [7]. Therefore, it is important to confirm that the inhibition of TLR4-mediated signaling by ethanol that has been reported in vitro and ex vivo also occurs when both ethanol and the TLR4 agonist are administered in vivo and the signaling events occur in vivo. In most cases, signaling cannot be evaluated using freshly isolated cells because of the transient nature of signaling and the time required to purify the desired cell type from the complex mixture of cells found in lymphoid organs, tissues, or blood. However, we reported previously that B6C3F1 mice have a population of resident cells in the peritoneal cavity that are >85% macrophages [8]. These cells can be obtained very quickly, placed on ice and extracted within a few minutes, thus allowing cellular signaling studies to be conducted using cells in which cellular signaling and any inhibition of that signaling actually occurred in the experimental animal. This approach is utilized here to quantitatively assess the effects of ethanol on signaling over time and cytokine production over time.

The mitogen activated protein (MAP) kinase pathway seems particularly important in TLR4 signaling [9], so key members of this group, ERK and p38, were evaluated. Among several transcription factors activated by TLR4 signaling, AP-1 and NF-κB are required for expression of several of the cytokines that are inhibited by ethanol (e.g., IL-6, TNF-α, and IL-12) [10-12]. Therefore, activation of these transcription factors was evaluated. Assessment of immunological parameters at only one point in time can yield results that are not at all representative of the overall effects over time. In the present study phoshphorylated (activated) ERK and p38 as well as phosphorylated c-Jun (a component of the AP-1 transcription factor) and nuclear p65 (as an indicator of NF-κB activation) were evaluated over the course of time and the area under the concentration vs time curve was calculated as an indicator of the overall difference in signaling over time. Similarly, cytokine concentrations in the peritoneal cavity were quantified over time. We have previously shown that IL-6 (as a representative pro-inflammatory cytokine) obtained by peritoneal lavage is not derived primarily from blood but produced locally [13], probably by the peritoneal macrophages in which signaling was evaluated.

The results generally confirmed previous reports of inhibition of signaling beginning early in the TLR4 signaling pathway and extending to the ends of the pathway, the activated transcription factors. However, there was no indication of inhibition of p65 translocation into the nucleus, suggesting that ethanol does not inhibit NF-κB activation. However, to insure that activation mediated by NF-κB proteins that do not contain p65 was also not inhibited, transgenic mice with an NK-κB luciferase reporter construct were evaluated by luminescence imaging in live anesthetized mice.

## Results

### Effects of ethanol on activation of MAP kinases, AP-1, and NF-κB

**Figure 1:**
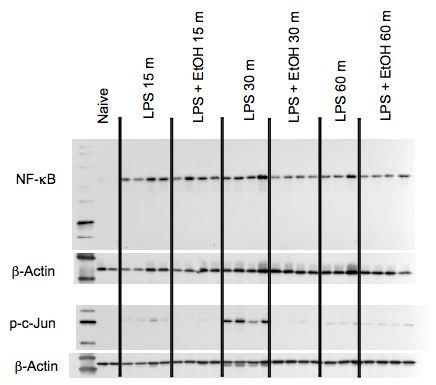
**Inhibition of nuclear translocation of transcription factors by ethanol**. Mice were treated with ethanol at 6 g/kg (by gavage with a 32%, v/v solution). Thirty minutes later, LPS (60 μg/mouse) was administered intravenously. Then at the indicated times after LPS administration, peritoneal macrophages were isolated by lavage using ice cold PBS. Nuclear extracts were prepared and the indicated proteins assessed by western blot. Each lane represents a sample from one mouse (n = 2 for naive; n = 4 for most groups; n = 3 for LPS + ethanol at 60 min). Quantitation and statistical analysis of these data are shown in Figures 2 and 3.

**Figure 2:**
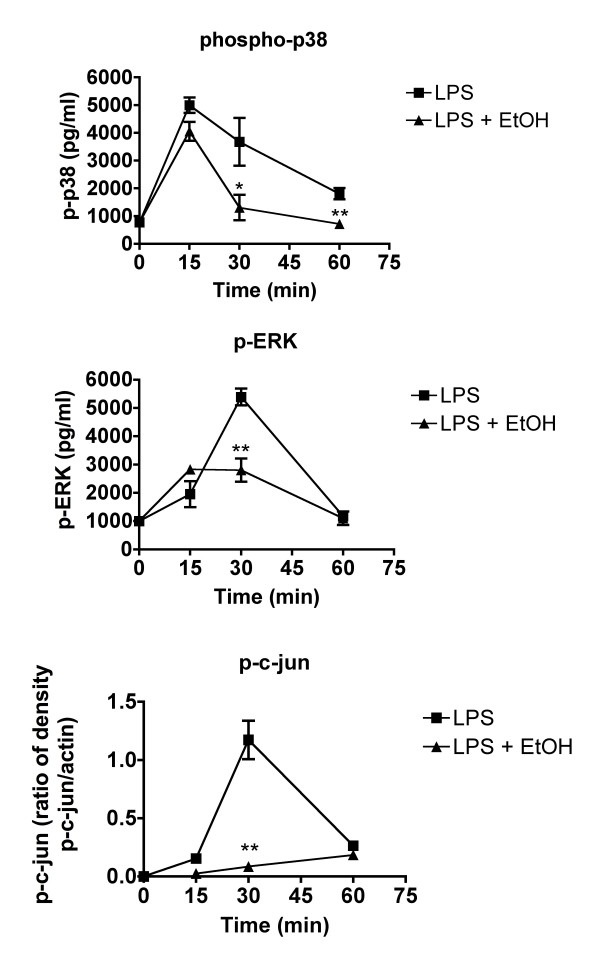
**Activation of signaling molecules induced by LPS *in vivo *is inhibited by ethanol**. Mice were treated with ethanol at 6 g/kg (by oral gavage with a 32%, v/v solution). Thirty minutes later, LPS (60 μg/mouse) was administered intravenously. Then at the indicated times after LPS administration, peritoneal macrophages were isolated by gavage using ice cold PBS. Cell pellets were immediately extracted and analyzed for the indicated phosphorylated proteins by western blot. Values shown are means ± SEM (n = 5 mice per group). Values at each time point for LPS-treated and LPS plus ethanol-treated animals were compared by Student's t test and significant differences are indicated by ** (p < 0.01).

In a previous study, we reported that ethanol alone at the same dosage and timing as used in the present study does not induce increases in the production of IL-6, IL-10, or IL-12 [13]. Thus, it seemed likely that "ethanol only" controls would not be informative, so they were not included. Data shown in Figure 1 indicate that activation of AP-1 (as indicated by phosphorylation and nuclear translocation of c-Jun, one of the components of AP-1) was suppressed by ethanol, and suppression was maximal at 30 min. Results shown in Figure 2 indicate that activation (as indicated by phosphorylation) of kinases upstream of these transcription factors were significantly inhibited and that the inhibition of p-c-Jun shown in Figure 1 was also significant at 30 min. Suppression was significant (by Student's t test) for at least one time point for all kinases, and suppression of p-p38 was almost significant at 30 min. Phosphorylation had decreased to unstimulated levels within 1 hr, indicating that the role of these signaling mediators was completed in that time and the area under the concentration vs. time curve in this study reflects the complete period of signaling. The area under the concentration vs. time curve values are shown in Table 1. As would be expected on the basis of the graphs in Figure 2, ethanol suppressed the AUC values in all cases. The activation of p-c-Jun was inhibited to a greater extent than p-p38 or p-ERK, and peritoneal cytokines were inhibited to varying degrees, but the ratio of LPS + ethanol to LPS groups was within the range of the values noted for signaling parameters, except that p-ERK was inhibited less than any of the cytokines in which ERK is involved in signaling. Assessing signaling in peritoneal macrophages and cytokines produced by these cells in the peritoneal cavity allows assessment of these parameters following a representative in vivo innate inflammatory response. The results are consistent with the idea that inhibition of signaling contributes to inhibition of cytokine production.

**Table 1:** AUC Values for signaling and cytokine production

	**LPS**	**LPS + Ethanol**	**Ratio**
p-p38	190694	106858	0.56
p-ERK	175677	129677	0.74
p-c-jun	33	5	0.15

IL-6	12885	4829	0.37
IL-12	1601	669	0.42
TNF-α	65	12	0.19
IL-1β	77	40	0.51

**Figure 3:**
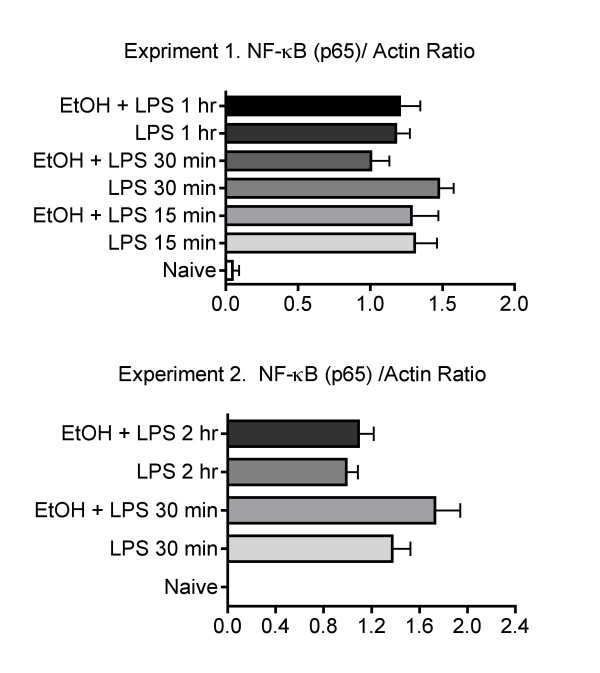
**Ethanol does not alter the translocation of the p65 component of NF-κB to the nucleus**. Nuclear extracts were prepared as described in materials and methods, and analyzed by western blot for p65. Values shown represent mean pixel density ± SEM (n = 5 mice per group). Values at each time point for LPS-treated and LPS plus ethanol-treated animals were compared by Student's t test and no significant differences were identified.

**Figure 4:**
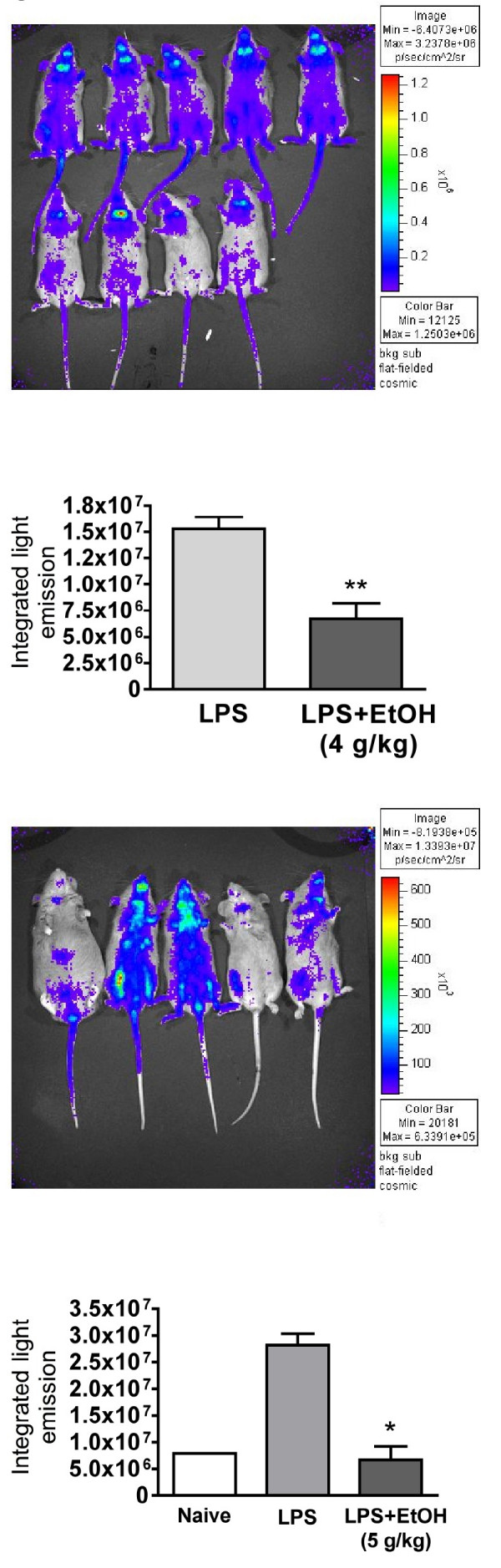
**Ethanol inhibits NF-κB reporter construct activation by LPS in vivo**. Transgenic mice with an NF-κB reporter driving expression of luciferase were imaged using an IVIS luminescence imager. Below each image of light emission profiles for the mice is a graph in which the data for those mice were analyzed using the IVIS software for integrated photon emission. Values shown are means ± SEM for the indicated group sizes. Values significantly different from the LPS control are indicated by ** (p < 0.01).

Results shown in Figure 3 indicate that LPS causes substantial activation of NF-κB, as indicated by translocation of p65 to the nucleus. Ethanol did not significantly inhibit this activation. However, it remained possible that other forms of NF-κB were inhibited by ethanol, so that overall inhibition of NF-κB could contribute to inhibition of the induction of cytokines. Therefore, transgenic mice with an NF-κB responsive luciferase reporter construct were used to detect activation of all transcription activating forms of NF-κB. Results shown in Figure 4 demonstrate significant inhibition of NF-κB activation by ethanol at 4 or 5 g/kg, which we have previously reported to produce peak blood ethanol levels of 200 and 300 mg% (43.5 and 65.2 mM), respectively [14].

### Effects of ethanol on cytokines in serum over time and dependence on time of ethanol administration

**Figure 5:**
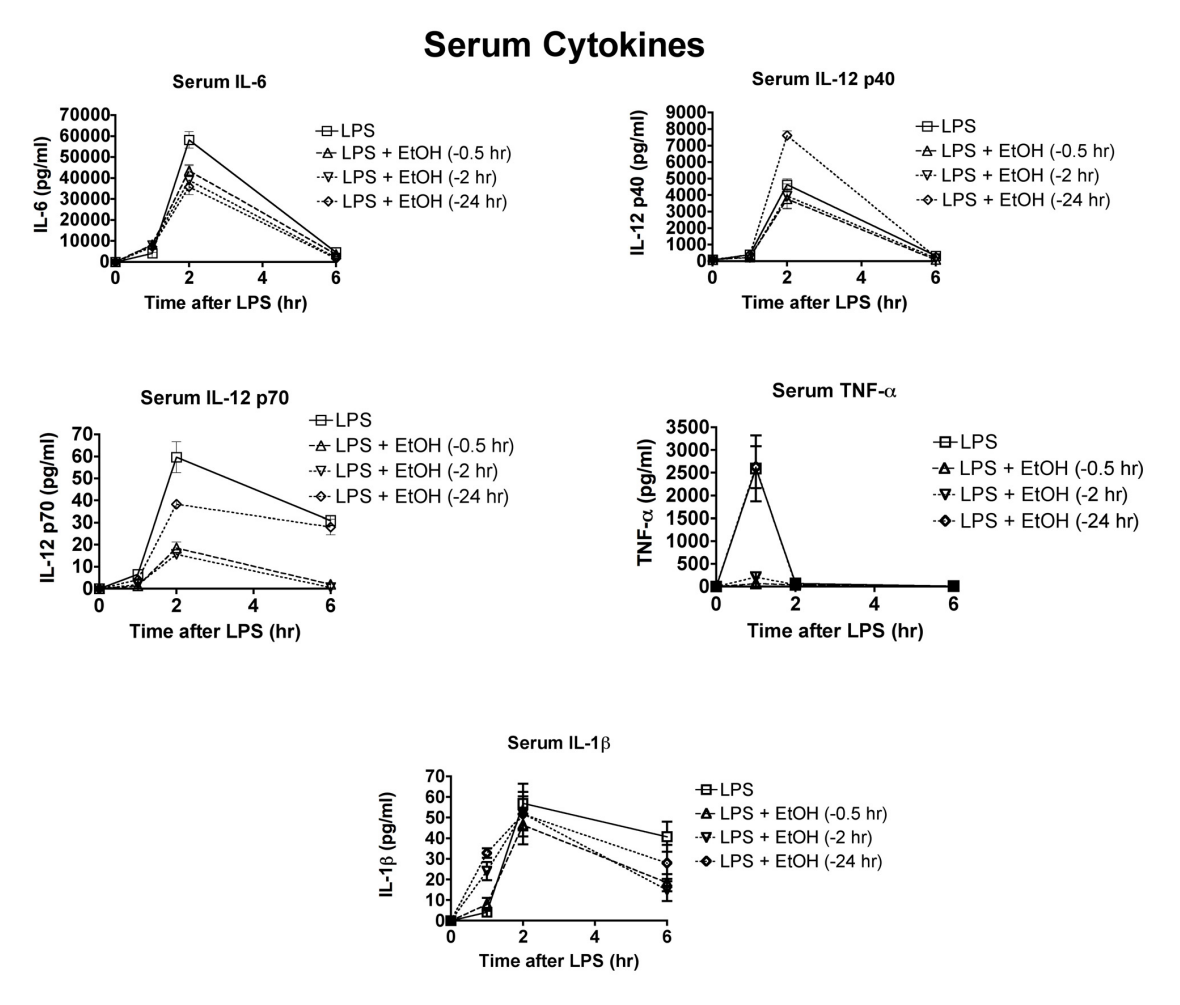
**Ethanol modulates production of some cytokines in serum, even when administered 24 hr before LPS**. Mice were treated with ethanol (6 g/kg by gavage) at the indicated times before LPS, and blood and peritoneal fluid samples were obtained for analysis 2 hr after LPS. Values shown are means ± SEM (n = 5). Because of overlapping values, showing statistical results on the graphs is not practical. Major statistically significant outcomes are identified in the text of the results section.

**Figure 6:**
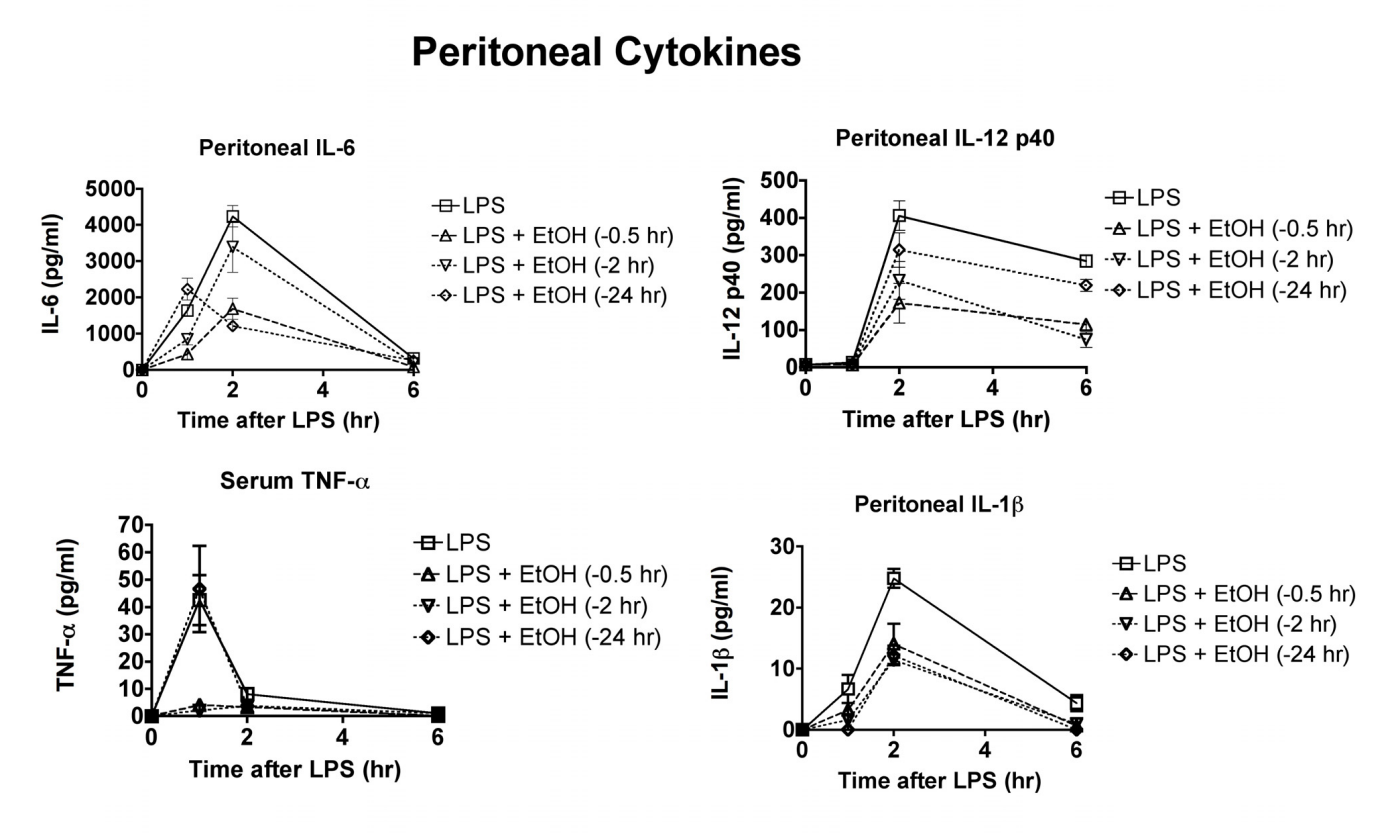
**Ethanol modulates production of some cytokines in peritoneal fluid even when administered 24 hr before LPS**. Mice were treated with ethanol (6 g/kg by gavage) at the indicated times before LPS, and blood and peritoneal fluid samples were obtained for analysis 2 hr after LPS. Values shown are means ± SEM (n = 5). Because of overlapping values, showing statistical results on the graphs is not practical. Major statistically significant outcomes are identified in the text of the results section.

Results shown in Figure 5 demonstrate that ethanol inhibits the production of most pro-inflammatory cytokines throughout the time that they are produced in response to LPS. The dosage used in this study (6 g/kg) yields a peak blood ethanol concentration of 400 mg% (87 mM) [15]. Such concentrations have been reported in binge drinkers, but more importantly, inhibition of cytokine production is dose responsive with effects noted at dosages as low as 3 g/kg, by gavage [16]. Because the duration of the ethanol action has not previously been determined in this model, ethanol was administered to different groups 24, 2, and 0.5 hr before administration of LPS (Figures 5 and 6). The concentration of IL-6 and IL-12 p70 in the serum was significantly decreased (p < 0.05) when ethanol was administered 24, 2, and 0.5 hr before LPS. This 24 hr duration of effects has not been reported previously and is relevant with regard to potential increased risk of infection not just on the day of binge drinking but possibly also the next day. The cytokines significantly suppressed by ethanol at one or more time points were IL-6, IL-12 p70, IL-12 p40, and TNF-α. The IL-1β response was also decreased, but the decrease was primarily at 1 hr after LPS. The concentration of IL-10 was significantly enhanced by treatment with ethanol 0.5 or 2 hr before LPS. A similar effect was noted in mice treated with polyinosinic polycytidylic acid to activate TLR3 [13]. One unexpected observation was the enhancement of IL-12 production by ethanol administration 24 hr before LPS. Previously such an increase had only been noted for IL-10 [13]. This increase was not observed for IL-12 p40, but it has been established that expression of the genes for IL-12 p40 and p35 are regulated independently and both of these proteins are required for the formation of the p70 heterodimer of IL-12 [17]. Thus, IL-12 p35 may be the limiting factor in IL-12 p70 production, and upregulation of IL-12 p35 could cause increased IL-12 p70 without an increase in IL-12 p40.

### Effects of ethanol on cytokines in the peritoneal cavity over time and dependence on time of ethanol administration

Previous studies indicate that serum and peritoneal cytokines are derived from different sources [13,18]. Because peritoneal macrophages were used in the cellular signaling studies reported here, it seemed appropriate to also assess cytokine production in the peritoneal cavity. Results of this analysis are shown in Figure 6. There were some clear differences in the pattern of ethanol effects on cytokine production as compared to effects on serum cytokines. For example, the peritoneal IL-1β concentration was decreased by ethanol administration at all three time points before LPS. The concentration of IL-6 was decreased less by administration of ethanol 0.5 hr before LPS than by administration 2 or 24 hr before. In contrast, all three times of administration yielded similar inhibition in the serum. The concentration of IL-12 p40 in the peritoneal cavity was decreased less when ethanol was administered 24 hr before LPS than at the other times of administration. In contrast, the concentrations in serum were similar for all three times of administration. The effects of ethanol at all times of administration were similar for TNF-α in serum and peritoneal fluid. It should be noted that the much lower concentration of cytokines in peritoneal lavage fluid than in serum may simply reflect the unavoidable dilution of the peritoneal fluid during the lavage process. The amount of dilution is not known, but it may be sufficient that the actual concentrations in the peritoneal cavity would be similar to those in serum.

## Discussion

Results shown in this study demonstrate that LPS-induced signaling through TLR4 is inhibited by ethanol in vivo in a mouse model for binge drinking. Rapid isolation of peritoneal macrophages allowed detection of the activation of all tested signaling parameters by LPS and any inhibition caused by ethanol. The mechanism by which ethanol disrupts signaling is not completely understood, but studies conducted primarily in vitro suggest that ethanol disrupts movement of components of the TLR4 receptor complex into lipid rafts and inhibits associated receptor clustering, rearrangement of the actin cytoskeleton, and activation of the signaling cascade [19-22]. The results presented here demonstrate that later signaling events are also inhibited by ethanol and that this occurs in vivo. Similar results were obtained by others following acute ethanol administration to mice in vivo and stimulation of cells with LPS ex vivo [23] and following acute ethanol exposure of human monocytes in culture [19]. Together, these results and the results reported here suggest that inhibition of production of pro-inflammatory cytokines by acute exposure to ethanol is a robust phenomenon that is probably mediated directly by ethanol's effect on ligand binding or events normally induced just after ligand binding to the TLR4 receptor complex.

The inhibition of signaling was greatest 30 min after LPS administration for ERK, p38, and c-Jun. This was the case even though the kinetics of activation were not all the same for these signaling molecules. This suggests that ethanol is most active with regard to signal disruption 1 hr after administration (30 min after LPS). This is near the time at which ethanol reaches maximum blood levels after intragastric administration of the dosage used in this study [14]. It was surprising that the LPS-induced translocation of the p65 component of NF-κB to the nucleus was not affected by ethanol. Most of the cytokines affected by ethanol require NF-κB for activation, so it would be surprising if inhibition of NF-κB activation could not be detected. Inhibition of LPS-induced p65 translocation has been reported in human monocytes treated with ethanol in vitro, but decreased phosphorylation of p65 (which was not assessed in the present study) was found to be more important [24]. It is not clear why no change in p65 translocation was observed here, but we have previously reported inhibition of p65 translocation by acute ethanol exposure in mice treated with polyinosinic polycytidylic acid to activate TLR3 [8]. The same methodology and materials were used in the present study, so it is unlikely the current negative result can be explained as a technical problem. However, the use of NF-κB reporter mice clearly demonstrated suppression of NF-κB activation. It is possible that p65 enters the nucleus normally in ethanol treated mice but decreased phosphorylation decreases its transactivation function. It is also possible that NF-κB composed of subunits other than p65 is responsible for activation of NF-κB mediated gene expression and that entry of these subunits into the nucleus is inhibited by ethanol. These issues are under investigation, and results will be reported in a separate publication.

The combination of decreased activation of AP-1 (as indicated by decreased phosphorylation of c-Jun) and NF-κB would be expected to inhibit LPS-induced expression of cytokines whose expression is dependent on these transcription factors. This includes TNF-α [12], IL-12 [25], IL-1β [26], and IL-6 [27]. All of these cytokines were significantly suppressed at one or more time points in the peritoneal cavity in ethanol-treated mice. The basis for the increased production of IL-10 is not clear, but it does not seem to involve a mechanism reported in human monocytes in which ethanol increases LPS-induced p38 activation, leading to increased heme oxygenase 1, which increases IL-10 gene expression [28]. In the present study, enhanced IL-10 production was associated with suppressed p38 activation. The basis for this difference is not clear, but this could either represent a species difference (human vs. mouse) or a difference in effects of LPS and/or ethanol in vivo (present study) vs. in vitro [28].

The decreased pro-inflammatory cytokines and increased IL-10 observed here would be expected to decrease resistance to infection, and we have previously reported such a decrease in mice treated by acute administration of ethanol [13]. Cytokines inhibited by ethanol in the present study, such as IL-12, IL-6, and TNF-α have been shown to be important in innate resistance to infections [29-31]. Increased IL-10 concentrations would be expected to decrease innate resistance to infections [32]. Furthermore, this decrease in some pro-inflammatory cytokines is observed even when ethanol is administered as much as 24 hr before immunological challenge. Thus, the time frame during which the risk of infection is increased might be at least 24 hr.

Differences were observed in the pattern of cytokine expression and effects of ethanol when comparing concentrations of cytokines in the serum and peritoneal cavity. For example, serum IL-1β was not inhibited significantly at 2 hr following LPS by ethanol administered 0.5, 2, or 24 hr before LPS. However, in the peritoneal cavity there was significant inhibition 2 hr after LPS caused by administration of ethanol at 0.5, 2, or 24 hr. Other differences were also noted, which are consistent with our previous conclusion that cytokines in the peritoneal cavity and in the serum are derived predominantly from different cells types which are affected differently both by LPS and ethanol [33]. Some of these differences could also be due to differences in the concentration, rate of clearance, and concentrations of active metabolites of ethanol in liver or spleen (sources for a substantial portion of pro-inflammatory cytokines in serum) as compared to peritoneal cavity. These issues are currently under investigation. In any case, the changes in signaling in peritoneal macrophages and changes in cytokine production in the peritoneal cavity are comparable, which is consistent with the idea that decreased signaling is a major cause of altered cytokine production.

The effects of ethanol at 0.5 or 2.0 hr would be consistent with our previous findings suggesting direct action of ethanol on macrophages, which requires that the ethanol is present during stimulation with LPS to produce the effect [21,22]. However, inhibition of the production of some cytokines by ethanol administered 24 hr before LPS demonstrates persistent effects as well. Ethanol is completely cleared in this model within 12 hr [14], so effects that persist 24 hr after dosing cannot represent direct action of ethanol, but must represent relatively persistent effects on cells. The mechanism for these effects is under investigation.

The results presented here indicate that activation of cellular signaling induced by LPS in peritoneal macrophages is inhibited by ethanol in vivo. The magnitude of changes in AUC values for signaling molecule activation and changes in AUC values for cytokine concentration were generally similar. Disparate amounts of change would not have supported a cause-effect relationship between inhibition of signaling and inhibition of cytokine production. However, the results shown here as well as abundant results from published studies indicating that signaling inhibitors decrease cytokine production [34-36], suggest that a cause-effect relationship between inhibition of signaling and inhibition of cytokine production is feasible. These results were obtained using an animal model in which ethanol has been demonstrated to decrease innate immunity to infection [13], suggesting that the observed changes are functionally relevant with regard to host resistance as well.

## Conclusion

The results shown here indicate that acute ethanol exposure inhibits signalling through TLR4 in vivo in a manner similar to that which has been demonstrated in vitro and ex vivo. However, detecting inhibition of NF-κB required the use of reporter mice and was not successful when p65 (the most common subunit of activating NF-κB heterodimers) was evaluated. The observation that ethanol given 24 hr before LPS inhibits production of some cytokines indicates a persistent effect of ethanol on cells such that inhibition of the response occurs even after ethanol is cleared.

## Methods

### Mice and treatments

Female C57Bl/6 × C3H F1 (B6C3F1) mice were obtained from Charles River Labs through the National Cancer Institute's animal program. Mice were allowed to recover from shipping stress for at least two weeks before use in experiments at 8-16 weeks of age. They were housed 5 per cage in a temperature and humidity controlled facility. Sentinel mice housed in the same room were negative for common mouse pathogens during the period of this study. Animal care and use was conducted in accord with the NIH Guide and the regulations of Mississippi State University. The animal care and use program at MSU is accredited by the American Association for Accreditation of Laboratory Animal Care. Ethanol was administered by gavage using a 32% solution (v/v) in tissue culture grade sterile water. This has been shown to yield blood ethanol concentrations relevant to binge drinking and not to cause necrosis or other gross histopathological changes in the gastrointestinal tract [14]. This model has been cited and used by a number of other investigators. Peritoneal macrophages were activated in vivo by intravenous administration of lipopolysaccharide (LPS, Sigma Chemical Co., St. Louis, MO, from *E. coli *0111:B4) at 60 μg/mouse.

### Evaluation of signalling

Cells for assessment of signaling were obtained at selected times after LPS administration by peritoneal lavage using 7 ml of ice cold phosphate buffered saline with 1% bovine serum albumin. The cell pellet from each mouse (after centrifugation at 300 × g for 5 min) was then used for analysis of signaling. Evaluation of p-c-Jun and NF-κB (p65) was done by preparing nuclear extracts as in our previous study [37] and using western blot analysis with antibodies to p-c-Jun and NF-κB p65 from Santa Cruz Biotechnology (Santa Cruz, CA). Secondary antibodies conjugated with horseradish peroxidase were obtained from the same source. Bands were visualized using a chemiluminescence kit (Amerasham, Chalfont St. Giles, UK). Images were obtained using a VersaDoc 3000 (BioRad, Hercules, CA). Relative quantitation of band intensities was accomplished as described previously using NIH Image software [37]. Actin was used as a loading control in these analyses. The quantity of phospho-p38 and phospho-ERK (p-p38 and p-ERK) was determined using a cell based assay kit (FACE, Active Motif, Carlsbad, CA).

### NF-κB reporter mice

Transgenic mice with a reporter construct consisting of a luciferase gene with an NF-κB dependent promoter were produced by Carlsen and colleagues [38] and obtained through Xenogen (Alameda, CA). Mice were treated with ethanol 30 min before stimulation of NF-κB activation. Mice were treated with bacterial lipopolysaccharide (from *E. coli *0128:B12, Sigma Chemical Co., catalog #L2755) by administering 60 μg/mouse, intravenously. Two hr after LPS, mice were anesthetized with 150 mg/kg ketamine and 5 mg/kg xyalzine intraperitoneally. Mice were then treated with luciferin (15 mg/kg, intraperitoneally), and imaging was done 5-10 min later using an IVIS imager (Xenogen, Alameda, CA).

### Evaluation of cytokine concentrations

Cytokines in serum and peritoneal lavage fluid were analyzed by multiplexed bead array analysis using a kit from BioRad and a Bioplex reader. Peritoneal lavage was done initially by removing the skin from the abdomen without puncturing the membrane of the peritoneal cavity then injecting 1 ml of ice cold phosphate buffered saline with 1% fetal bovine serum. The abdomen was massaged to dislodge cells and mix, and a 25 g needle and syringe was used remove the fluid (usually 0.7 ml of the original 1 ml was obtained). Cytokines were assessed at various times after challenge with LPS (indicated in figures).

### Data analysis and statistics

Area under the curve values for cytokine expression over time and activation of signaling molecules over time were calculated by entering the data in Prism 4.0 software (GraphPad, LaJolla, CA) and selecting area under the curve in the analysis menu. Statistical comparisons were done by Analysis of Variance (ANOVA) or Student's t test (to compare 2 groups) at each time point, using Prism 4.0 software (GraphPad Software, LaJolla, CA).

## Authors' contributions

SBP designed the study, identified appropriate methods, helped with harvesting blood and peritoneal fluid, and wrote the manuscript. RF performed most of the experimental procedures and edited the manuscript. Both authors reviewed and approved this manuscript.
